# Human serum fetuin A/α2HS-glycoprotein level is associated with long-term survival in patients with alcoholic liver cirrhosis, comparison with the Child-Pugh and MELD scores

**DOI:** 10.1186/1471-230X-7-15

**Published:** 2007-03-29

**Authors:** László Kalabay, László Gráf, Krisztián Vörös, László Jakab, Zsuzsa Benkő, László Telegdy, Béla Fekete, Zoltán Prohászka, George Füst

**Affiliations:** 1 Department of Family Medicine, Semmelweis University, Kútvölgyi út 4, 1125 Budapest, Hungary; 2 3rd Department of Medicine Semmelweis University, Kútvölgyi út 4, 1125 Budapest, Hungary; 3 3^rd ^Department of Internal Medicine, Szt. László Hospital, Gyáli út 5–7, 1097 Budapest, Hungary; 4 Research Group of Metabolism, Genetics and Immunology, Hungarian Academy of Sciences, Kútvölgyi út 4, 1125 Budapest, Hungary

## Abstract

**Background:**

Serum concentration of fetuin A/α2HS-glycoprotein (AHSG) is a good indicator of liver cell function and 1-month mortality in patients with alcoholic liver cirrhosis and liver cancer. We intended to determine whether decreased serum AHSG levels are associated with long-term mortality and whether the follow-up of serum AHSG levels can add to the predictive value of the Child-Pugh (CP) and MELD scores.

**Methods:**

We determined serum AHSG concentrations in 89 patients by radial immunodiffusion. Samples were taken at the time of enrolment and in the 1^st^, 3^rd^, 6^th^, and the 12^th ^month thereafter.

**Results:**

Forty-one patients died during the 1-year follow-up period, 37 of them had liver failure. Data of these patients were analysed further. Deceased patients had lower baseline AHSG levels than the 52 patients who survived (293 ± 77 vs. 490 ± 106 μg/ml, mean ± SD, p < 0.001). Of all laboratory parameters serum AHSG level, CP and MELD scores showed the greatest difference between deceased and survived patients. The cutoff AHSG level 365 μg/ml could differentiate between deceased and survived patients (AUC: 0.937 ± 0.025, p < 0.001, sensitivity: 0.865, specificity: 0.942) better than the MELD score of 20 (AUC: 0.739 ± 0.052, p < 0.001, sensitivity: 0.595, specificity: 0.729). Initial AHSG concentrations < 365 μg/ml were associated with high mortality rate (91.4%, relative risk: 9.874, 95% C.I.: 4.258–22.898, p < 0.001) compared to those with ≥ 365 μg/ml (9.3%). Fourteen out of these 37 fatalities occurred during the first month of observation. During months 1–12 low AHSG concentration proved to be a strong indicator of mortality (relative risk: 9.257, 95% C.I.: 3.945–21.724, p < 0.001). Multiple logistic regression analysis indicated that decrease of serum AHSG concentration was independent of all variables that differed between survived and deceased patients during univariate analysis. Multivariate analysis showed that correlation of low serum AHSG levels with mortality was stronger than that with CP and MELD scores. Patients with AHSG < 365 μg/ml had significantly shortened survival both in groups with MELD < 20 and MELD ≥ 20 (p < 0.0001 and p = 0.0014, respectively).

**Conclusion:**

Serum AHSG concentration is a reliable and sensitive indicator of 1-year mortality in patients with alcoholic liver cirrhosis that compares well to the predictive value of CP score and may further improve that of MELD score.

## Background

Human fetuin A/α2HS-glycoprotein (AHSG) is a serum glycoprotein with a concentration around 450–600 μg/ml in healthy persons. AHSG is a negative acute phase reactant [1], accumulates in bone tissue and prevents untoward calcification [2,3]. Mice knocked-out for AHS gene exhibit extraosseal calcification [4]. AHSG is a natural antagonist of growth factors like insulin [5,6], transforming growth factor β (TGF-β) [7], hepatocyte growth factor/scatter factor (HGF/SF) [8] and inhibits lymphocyte blastic transformation [9]. These findings indicate that AHSG may be involved in tissue regeneration [8].

Since serum AHSG is produced exclusively by hepatocytes in adults [10] and its concentration decreases during the acute phase reaction we investigated its changes in patients with liver diseases [11,12]. We found low serum AHSG levels in patients with acute alcoholic hepatitis, acute drug-induced hepatitis, chronic autoimmune hepatitis, fatty liver, alcoholic and primary biliary cirrhosis, and hepatocellular cancer [11-13]. Alterations were most marked in patients with alcoholic liver cirrhosis and hepatocellular cancer [11,12]. In these patients short-term (i.e. for one month) follow-up indicated that serum concentration of AHSG is an excellent indicator of liver function and mortality [11]. Serum AHSG levels showed by far the greatest difference when we compared laboratory parameters of deceased and survived patients with liver cirrhosis and cancer. Decreased levels (≤ 300 μg/ml) were associated with high mortality rate (52%, relative risk: 5.497, p < 0.0001). In this respect serum AHSG was the best of all laboratory parameters studied. Multiple logistic regression analysis indicated that serum AHSG level was independent of all parameters that were pathologic in deceased patients [11].

In this study we tried to determine the usefulness of serum AHSG levels for the long-term (i.e. up to 1 year) follow-up of patients with alcoholic liver cirrhosis. We intended to compare the predictive value of serum AHSG levels to those of the Child-Pugh (CP) and the Model for End-Stage Liver Disease (MELD) scores. Both scores have been widely used to estimate the outcome of patients with end-stage liver disease, including alcoholic cirrhosis [14-16]. Herein we report that low serum AHSG concentration is a reliable and sensitive indicator of 1-year mortality in patients with alcoholic liver cirrhosis, comparing to the CP score and may further improve the predictive value of the MELD score.

## Methods

### Patients

Ninety-three patients with alcoholic liver cirrhosis (52 men, 41 women, mean age: 54 ± 13 years, mean ± SD) were tested. They gave written consent approved by the Ethical Committee of Semmelweis University. Besides history and appropriate clinical symptoms the diagnosis was established by abdominal ultrasonography, and liver scan. Exclusion criteria were as follows: cirrhosis of viral origin (HBsAg and anti-HCV positivity), or autoimmune aetiology (antinuclear and anti-smooth muscle antibodies), liver cancer, and treatment with hepatotoxic drugs. For ethical reasons liver biopsy was not performed. Blood samples were taken at the time of enrolment and in the 1^st^, 3^rd^, 6^th ^and 12^th ^months thereafter. Patients with alcoholic liver cirrhosis were only on supportive therapy (diuretics, beta-blockers).

The MELD score was calculated according to the formula of Kamath et al. (3.8 * ln serum bilirubine [mg/dl] + 11.2 * ln INR + 9.6 * ln serum creatinine [mg/dl] + 6.4) [16]. The CP score of patients was calculated by rating the following parameters between 1 and 3: ascites, encephalopathy, prothrombin time (< 15, 15–17, > 17s), serum bilirubin (< 2, 2–3, > 3 mg/dl), serum albumin (> 35, 28–35, < 28 g/l) [14,15]. There were 53 patients with grade 1, 20 with grade 2 and 16 with grade 3 encephalopathy, 27 patients with grade 1, 36 with grade 2, and 26 with grade 3 ascites, and 16 patients with CP score 5–6, 46 with 7–9, and 30 with 10 or higher, respectively.

### Determination of serum AHSG concentration

Serum levels of AHSG were determined by radial immunodiffusion (RID) using 10 × 10 cm slides as described previously [11]. In brief, 5 μl of patient's sera diluted to 1:4 was applied in 11.5 ml of Litex agarose gel (Sigma). Serum samples (1:4 dilution) with known concentrations of AHSG served as standards. The incubation was done at room temperature for 48 hours. We used anti-AHSG (IgG fraction, Incstar, Cat No. 81931, 13.7 mg/ml, in a final concentration of 84 μl/11.5 ml gel) as antibody. The variation coefficient of the test was 3.6%.

### Other laboratory determinations

The determination of the erythrocyte sedimentation rate (ESR), hematocrit, hemoglobin, white blood cell (WBC), granulocyte and platelet (PLT) count, serum bilirubin, serum aspartate aminotransferase (ASAT), alanine aminotransferase (ALAT), alkaline phosphatase activities, International Normalisation Rate (INR), total serum protein and albumin concentrations was performed using conventional standardized methods. Serum levels of α2-macroglobulin, transferrin (TF), haptoglobin, and α1-acid glycoprotein/orosomucoid (OROSO) were measured by RID. Variation coefficients were 5.0%, and 4.2% for OROSO and TF measurements, respectively. C-reactive protein (CRP) concentration was determined by a particle-enhanced immunoturbidimetric assay (Roche Cobas Integra 4000^®^). The detection limit of the assay was 0.07 mg/l, the coefficient of variation was 3.9% at 108 mg/l mean value.

### Statistical analysis

Paired and unpaired data were analysed by the Wilcoxon and Mann-Whitney U tests, respectively. Correlation studies were done by the rank correlation analysis (Spearman). Contingency tables were analysed by the Fisher's exact test. Patients' survival was calculated by the Kaplan-Meyer test and life table analysis. These tests, the multiple logistic regression, and the receiver operating characteristic (ROC) curve analyses were performed with the SPSS v.11.5 statistical program. The level of p < 0.01 was considered significant.

## Results

### Relationship between initial serum AHSG concentration and 1-year survival of patients with alcoholic liver cirrhosis

During the one-year follow up 41 out of the 93 patients died. Four patients died of intercurrent illnesses unrelated to liver disease (2 of acute left ventricular failure, 1 of myocardial infarction, and 1 of pneumonia, respectively). The remaining 37 patients died of liver failure, and their data were analysed further. Table 1*Column A *shows the laboratory data of patients according to the outcome at the end of the one-year follow-up period. Compared to survivors we found significantly lower baseline values of hematocrit, albumin, TF and AHSG concentrations and significantly higher creatinine, bilirubine, INR, CP and MELD score values in deceased patients. Serum AHSG level showed one of the greatest differences among all laboratory parameters tested. We observed significantly positive correlations of AHSG with hematocrit (r = 0.406, p= 0.001), albumin (r = 0.366, p = 0.002), TF (r = 0.529, p < 0.001), and negative correlations with serum creatinine (r = -0.312, p = 0.006), INR (r = -0.467, p < 0.001), CRP (r = -0.405, p = 0.001), the MELD (r = -0.342, p = 0.001) and CP scores (r = -0.522, p < 0.001) whereas no significant correlations were found with the other parameters tested.

### Determination of the predictive accuracy of serum AHSG concentration

We used the ROC curve analysis to determine the optimal cutoff value and test the discrimination ability of serum AHSG concentration (Table 2, *Column A*). Based on this curve, the AHSG concentration of 365 μg/ml has been identified as the optimal cutoff value for stratification of our patients. At 365 μg/ml the relative risk, sensitivity and specificity were higher than those of the cutoff value 300 μg/ml, which was used at the short-term study.

**Figure 1:**
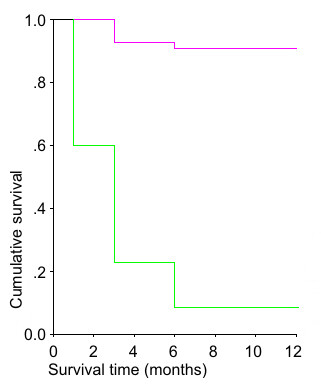
**Patients with serum AHSG concentration < 365 μg/ml have lower survival rate than those with ≥ 365 μg/ml throughout the 1-year observation period**. Serum AHSG concentration < 365 μg/ml (green line, n = 35), serum AHSG concentration ≥ 365 μg/ml (magenta line, n = 54), log rank test, p < 0.001

Initial AHSG levels below 365 μg/ml were associated with a significantly higher risk of mortality than those with values above this level (Table 3A). This finding was confirmed by the Kaplan-Meyer analysis of patients' survival (Figure 1). The calculated mean survival time of patients with AHSG levels below and above 365 μg/ml was 3 ± 1 months (mean ± S.E., 95% C.I.: 2 – 4) and 11 ± 1 months (95% C.I.: 11 – 12, p < 0.0001), respectively (log rank analysis). Life table analysis resulted in a median survival of 3.27 months in the former and over 12 months in the latter group, respectively (p < 0.0001). The hazard rate of fatal outcome increased with time in patients with serum AHSG < 365 μg/ml but not in those with higher AHSG levels (Table 4).

There were 6 patients with concomitant alcoholic hepatitis (serum AHSG: 405 ± 85 μg/ml, mean ± S.D.), whose values did not differ significantly from those without it (434 ± μg/ml, n = 83, p = 0.447). In patients with alcoholic hepatitis baseline AHSG increased to 454 ± 101 μg/ml (p = 0.500). The statistical comparison (Wilcoxon test), however, is not relevant due to small sample size. Seven patients out of the 89 with alcoholic cirrhosis continued to drink. Their serum AHSG level (259 ± 97 μg/ml, mean ± S.D.) was significantly lower than those who were abstinent (418 ± 129 μg/ml, n = 82, p = 0.003). Six of them died during the observation period. The only one survivor had baseline AHSG concentration above 365 μg/ml.

On enrolment 4 patients had infection: one spontaneous bacterial peritonitis, one cholangitis, one erysipelas, and one uroinfection, respectively. Their mean baseline serum AHSG level (445 ± 126 μg/ml, mean ± SD) increased to 475 ± 171 μg/ml by the time the next sample was taken (Wilcoxon test, p = 0.465).

**Figure 2:**
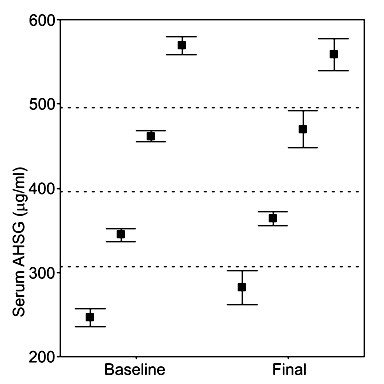
**Serum AHSG concentration did not change considerably during the 1-year observation period**. Values represent mean ± SEM. Comparison was performed by the Wilcoxon test. Baseline values of patients who died during the first month of observation (n = 14) were omitted from the analysis.

Serum AHSG levels did not vary considerably throughout the entire observation period as it is illustrated in Figure 2. Patients (n = 75) were divided to four quartiles according to their baseline AHSG levels. Patients in these four quartiles were followed and their final AHSG levels were compared to their baseline quartile values. Final AHSG values were within the range of the baseline quartiles (significance of difference between baseline and final values in quartiles 1, 2, 3, and 4 were p = 0.022, p = 0.248, p = 0.897, and p = 0.126, respectively). Patients with baseline values below 300 μg/ml almost never reached values above 350 μg/ml, and vice versa.

### Serum AHSG concentration is an independent predictor of long-term mortality

Low serum AHSG concentration was associated with high mortality in patients studied. Comparison of laboratory data of deceased and survived patients yielded significant differences in the MELD score (including creatinine, INR), hematocrit, albumin, TF, and AHSG values (Table 1). We used multiple logistic regression analysis in order to adjust for the effect of these confounding variables (Table 5A). Significant regression coefficient between AHSG levels and 1-year mortality was found even after adjustment for the confounding parameters listed above both in the model including creatinine and INR and in the one using MELD instead.

### Analysis of survival between months 1 and 12

We have previously demonstrated that low serum AHSG levels are associated high mortality during one-month follow-up period [11]. As Figure 2 shows 14 out the 37 fatalities occurred in the first month. In order to focus on the utility of AHSG levels between the 1^st ^and the 12^th ^months we re-run our analyses following exclusion of data of patients who died in the first month.

Only creatinine, INR, albumin, TF, AHSG levels, the CP and MELD scores differed significantly between deceased and survived patients (Table 1, Column B). In this observation period only hematocrit (r = 0.368, p = 0.004), INR (r = -0.404, p < 0.001), albumin (r = 0.331, p = 0.007), TF (r = 0.499, p < 0.001), the MELD (r = -0.271, p = 0.018) and CP (r = -0.431, p < 0.001) scores correlated with AHSG values significantly. Low AHSG levels were still associated with an elevated mortality rate and a high relative risk to die after the first month of observation (Table 3B).

**Figure 3:**
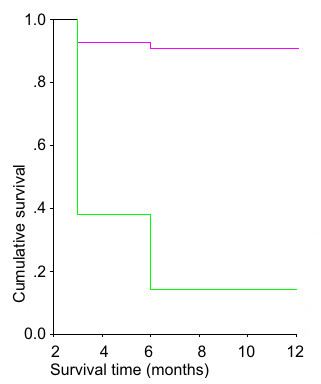
**Patients with serum AHSG concentration < 365 μg/ml have lower survival rate than those with ≥ 365 μg/ml between months 1–12**. Serum AHSG concentration < 365 μg/ml (green line, n = 21), serum AHSG concentration ≥ 365 μg/ml (magenta line, n = 54), log rank test p < 0.001

The Kaplan-Meyer test showed a strong difference between survivals of patients with low or high initial AHSG values (Figure 3). The calculated mean survival time with AHSG levels below and above 365 μg/ml was 5 ± 1 months (mean ± S.E., 95% C.I.: 4 – 6) and 11 ± 0 months (95% C.I.: 11 – 12, p < 0.001), respectively (log rank analysis). Life table analysis resulted in a median survival of 4.81 months in the former and over 12 months in the latter group, respectively (p = 0.001).

The association between low serum AHSG level and 1–12-month mortality was further analysed by a multiple logistic regression, in which data were adjusted for the confounding variables that significantly differed between deceased and survived patients (hematocrit, creatinine, INR, albumin, TF). AHSG levels had significant p values in the model using creatinine and INR and in the one using MELD instead. When data were adjusted for the confounding variables a significant regression coefficient was still found between AHSG levels and 1–12 month mortality both in the model with creatinine and INR and in the one with MELD (Table 5*Column B*).

### Comparison of the predictive accuracies of the CP, MELD scores and serum AHSG concentration

On ROC curve analysis the area under curve (AUC) of serum AHSG exceeded those of the CP and MELD scores (Table 2). The sensitivity and specificity of these cutoff AHSG values were still above those of CP and MELD.

In accordance with most observations we stratified our patients at the CP score of 10 [14,15]. The MELD score value ≥20 is generally accepted as an indication for liver transplantation [16]. Therefore we stratified MELD at 20. Patients with MELD ≥ 20 had higher relative risk to die within a year than those with smaller values but this risk was below what we found for AHSG < 365 μg/ml (Table 2). The risk of lethal outcome in patients with CP < 10 was comparable with those of low baseline AHSG values.

**Figure 4:**
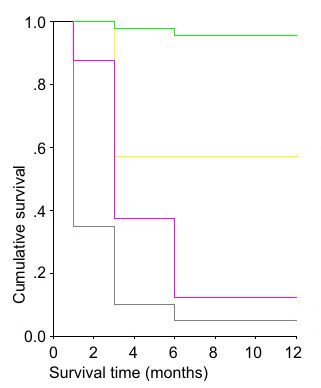
**The joint effect of serum AHSG concentration and the MELD score on the survival of patients with alcoholic liver cirrhosis**. Group 1 (green line): MELD < 20, serum AHSG ≥ 365 μg/ml (n = 46), group 2 (magenta line): MELD < 20, serum AHSG < 365 μg/ml (n = 16), group 3 (yellow line): MELD ≥ 20, serum AHSG ≥ 365 μg/ml (n = 7), and group 4 (grey line): MELD ≥ 20, serum AHSG < 365 μg/ml (n = 20), respectively. Compared to group 1, the survival is significantly shortened in groups 2, 3, and 4. Compared to group 1 the Odds Ratios for lethal outcome in groups 2, 3, and 4 are: 157.5 (95% C.I.: 20.28 – 1223, p < 0.001), 16.875 (95% C.I.: 2.149 – 132.5, p = 0.012), and 405.0, 95% C.I.: 34.53 – 4749 p < 0.0001), respectively.

Figure 4 illustrates the significant difference between survival of patients with MELD < 20 or ≥ 20 and serum AHSG > or ≥ 365 μg/ml. In order to analyse the joint effect of MELD and serum AHSG on survival of patients with liver cirrhosis patients were divided to four groups according to these critical values. Compared to group 1 (MELD < 20 and serum AHSG < 365 μg/ml) group 2 (MELD < 20 and serum AHSG ≦ 365 μg/ml) had a significantly shortened survival: 5 ± 1 months, 95% C.I.: 3–6 months vs. 12 months, 95% C.I. 11 – 12 months (log rank analysis: p < 0.001). The survival of patients in group 3 (MELD ≥ 20 and serum AHSG ≥ 365 μg/ml) was also reduced (mean survival time: 8 ± 2 months, 95% C.I.: 5 – 11 months, log rank analysis: p = 0.008. Group 4 (MELD ≥ 20 and serum AHSG < 365 μg/ml) had the worst prognosis: mean survival time: 2 ± 1 months (95% C.I.: 1 – 3 months, log rank analysis: p = 0.00001. Survival between groups 3 and 4 also differed significantly (p = 0.001).

Finally we set up a multiple logistic regression model to compare the predictive values of serum AHSG concentration to those of the CTP and MELD scores (Table 6). Serum AHSG values had the strongest association with mortality.

## Discussion

Previously we found serum AHSG to be an outstanding laboratory parameter of liver function an independent predictor of short-term mortality during the 1-month follow-up period [11]. Data gained during the first month of the present study supported our previous observations on short-term survival. Our present data suggest that the initially low serum AHSG concentration has an impact on long-term mortality as well. Excluding fatal outcomes that occurred during the first month survival analysis showed that low serum AHSG levels were still markedly associated with mortality. The marked difference of mean survival time between patients with low and normal AHSG levels indicated by the Kaplan-Meyer and life table analysis were in complete accordance with these observations.

The proinflammatory cytokines interleukin (IL)-1, IL-6, and tumour necrosis factor-α (TNFα) are known to enhance the synthesis of the positive (CRP, OROSO) and down-regulate that of the negative acute phase reactants in rat liver and human HepG2 cells [17,18]. Our short-term follow-up results suggested that the decreased serum AHSG concentration was associated rather with hepatocyte dysfunction than with the acute phase reaction [11]. During the 1-year follow up, a strong positive correlation with the negative acute phase protein TF and a negative correlation with the positive acute phase protein CRP have been observed. This latter correlation, however, was not significant between months 1–12. We found no significant correlation with the positive acute phase reactant OROSO. This suggests that the acute phase reaction is neither a determinant of the concentration of these proteins nor affects the long-term outcome in patients with liver cirrhosis. Thus, in accordance with our earlier findings low serum AHSG concentration reflects rather hepatocyte dysfunction than the acute phase reaction [11].

It has to be mentioned, however, that serum AHSG concentration is not decreased in every liver disease. There is no change in acute A, B, or Epstein-Barr virus hepatitis, and, moreover, there is a paradoxic elevation of AHSG levels in sera of patients with chronic C virus hepatitis [11,12,19]. Therefore serum AHSG concentration has probably no prognostic significance in these diseases.

Since its introduction in 2001 the MELD score has been used widely to predict the outcome of patients with end-stage liver disease [16]. The advantages and disadvantages of MELD and its comparison to the classical CP score have been reviewed extensively [14,15]. Though the clinical usefulness of MELD is established well modifications have been still recommended [20]. We have also found that CP performs slightly better than MELD. Logistic regression studies indicate that the CP, MELD and serum AHSG levels predict mortality somewhat independently from each other.

## Conclusion

The sensitivity, specificity and predictive values of serum AHSG concentration exceed those of the CP and the MELD scores. Although not in wide use the measurement serum AHSG concentration is simple. Serum AHSG levels may add to the power of MELD. A study performed on a large series of patients is needed to decide whether the combination of these parameters will result in an improved predictive value, and if so, how serum AHSG levels could be formulated.

## Competing interests

The author(s) declare that they have no competing interests.

## Authors' contributions

The authors contributed equally to this work. LK conceived of the study and determination of serum AHSG concentration and prepared the manuscript. LG and GF participated in the study design. LG, LJ, KV, ZB, LT and BF followed the patients and provided their clinical and laboratory data. ZP carried out the statistical analysis, GF gave critical reviews of the manuscript repeatedly. All authors read and approved the final manuscript.

## Pre-publication history

The pre-publication history for this paper can be accessed here:



## Figures and Tables

**Table 1:** Comparison of laboratory parameters of deceased and survived patients with alcoholic liver cirrhosis during the one-year follow-up period (Column A) and between months 1 and 12 (Column B)

	**(A) 1-year follow-up period**			**(B) Follow-up between months 1–12**		
**Parameter**	**Deceased patients mean ± SD n = 37**	**Survived patients mean ± SD n = 52**	**p**	**Deceased patients mean ± SD n = 23**	**Survived patients mean ± SD n = 52**	**p**

Age, years	57 ± 14	52 ± 12	0.056	58 ± 15	52 ± 12	0.056
Erythrocyte sedimentation rate, mm/h	57 ± 32	40 ± 30	0.019	59 ± 34	40 ± 30	0.030
Hematocrit, l/l	0.32 ± 0.1	0.37 ± 0.1	0.004	0.33 ± 0.1	0.36 ± 0.1	0.029
Hemoglobin, g/l	112 ± 18	121 ± 24	0.053	113 ± 18	121 ± 24	0.137
White blood cell count, μl^-1^	9582 ± 5076	7706 ± 4640	0.016	8960 ± 5102	7706 ± 4639	0.082
Granulocyte count, μl^-1^	6308 ± 4080	4788 ± 3241	0.080	5409 ± 3253	4787 ± 3240	0.311
Platelet count, μl^-1^	16061 ± 117289	177370 ± 91742	0.228	153480 ± 114813	177370 ± 91742	0.185
Serum creatinine, mg/dl	1.31 ± 0.65	0.96 ± 0.29	0.002	1.32 ± 0.62	0.97 ± 0.28	0.001
Serum bilirubin, mg/dl	10.7 ± 1.0	6.90 ± 10.1	0.008	7.8 ± 1.3	7.13 ± 171	0.041
Serum aspartate aminotransferase, U/l	113 ± 80	122 ± 171	0.201	107 ± 81	122 ± 10	0.485
Serum alanine aminotransferase, U/l	79 ± 137	143 ± 351	0.852	97 ± 173	142 ± 350	0.763
Alkaline phosphatase, U/l	425 ± 422	371 ± 284	0.987	404 ± 479	371 ± 283	0.571
INR	1.73 ± 0.7	1.25 ± 0.4	< 0.001	1.64 ± 0.7	1.26 ± 0.4	< 0.001
Total protein, g/l	63 ± 7	68 ± 10	0.453	68 ± 7	68 ± 9	0.815
Albumin, g/l	31 ± 7	37 ± 6	0.001	31 ± 7	36 ± 7	0.008
CRP, mg/l	62 ± 78	42 ± 78	0.061	65 ± 103	43 ± 79	0.505
α2-macroglobulin, mg/dl	315 ± 87	339 ± 123	0.588	335 ± 97	340 ± 123	0.851
Haptoglobin, mg/dl	186 ± 260	201 ± 150	0.035	203 ± 280	200 ± 151	0.108
Transferrin, mg/dl	148 ± 52	216 ± 86	< 0.001	148 ± 60	217 ± 87	0.003
α1-acid glycoprotein, mg/dl	94 ± 51	112 ± 53	0.049	93 ± 54	112 ± 54	0.076
CP score	11 ± 2	7 ± 2	< 0.001	11 ± 2	8 ± 2	< 0.001
MELD score	20 ± 9	13 ± 5	< 0.001	20 ± 8	13 ± 6	< 0.001
AHSG, μg/ml	293 ± 77	490 ± 106	< 0.001	303 ± 91	490 ± 91	< 0.001

p: significance calculated by the Mann-Whitney test

**Table 2:** Comparison of the sensitivity, specificity and predictive values of CP, MELD scores and serum AHSG in patients with alcoholic liver cirrhosis

	**(A) 1-year follow-up period**	**(B) Follow-up between months 1–12**
**CP score**		
AUC ± SD	0.865 ± 0.040	0.855 ± 0.050
p	< 0.001	< 0.001
95% C.I.	0.787 – 0.943	0.757 – 0.953
**MELD score**		
AUC ± SD	0.739 ± 0.052	0.740 ± 0.058
p	< 0.001	0.001
95% C.I.	0.637 – 0.871	0.626 – 0.854
**Serum AHSG**		
AUC ± SD	0.937 ± 0.025	0.908 ± 0.035
p	< 0.001	0.001
95% C.I.	0.889 – 0.986	0.839 – 0.978
**CP score < 10, ≥ 10**		
Relative Risk	3.878	4.838
p	< 0.001	< 0.001
95% C.I.	1.998 – 7.527	2.009 – 11.649
Sensitivity at this cutoff value	0.753	0.675
Specificity at this cutoff value	0.827	0.755
**MELD score < 20, ≥ 20**		
Relative Risk	2.205	2.4167
p	0.002	0.011
95% C.I.	1.318 – 3.689	1.229 – 4.952
Sensitivity at this cutoff value	0.595	0.587
Specificity at this cutoff value	0.729	0.720
**AHSG < 365 μg/ml, ≥ 365 μg/ml**		
Relative Risk	9.874	9.257
p	< 0.001	< 0.001
95% C.I.	4.258 – 22.898	3.945 – 21.724
Sensitivity at this cutoff value	0.865	0.783
Specificity at this cutoff value	0.942	0.936
**AHSG < 300 μg/ml, ≥ 300 μg/ml**		
Relative Risk	4.209	4.813
p	< 0.001	< 0.001
95% C.I.	2.675 – 6.622	2.813 – 8.233
Sensitivity at this cutoff value	0.594	0.478
Specificity at this cutoff value	0.981	0.972

**Table 3:** Effect of initial serum AHSG level on the survival of patients with alcoholic liver cirrhosis throughout the 1-year observation period (A) and between months 1–12 (B)

**A**				
	**Deceased**	**Survived**	**Total**	**Mortality rate %**

Serum AHSG < 365 μg/ml	32	3	35	94.1
Serum AHSG ≥ 365 μg/ml	5	49	54	9.3
Total	37	52	89	
p < 0.001, Relative Risk: 9.874 (95% C.I.: 4.258 – 22.898)				

**B**				

	**Deceased**	**Survived**	**Total**	**Mortality rate %**

Serum AHSG < 365 μg/ml	18	3	21	85.7
Serum AHSG ≥ 365 μg/ml	5	49	54	9.3
Total	23	52	75	
p < 0.001, Relative Risk: 9.257 (95% C.I.: 3.945 – 21.724)				

**Table 4:** Fatality risk over the one-year observation period in patients with serum AHSG levels above and below 365 μg/ml

**Hazard rate at**	**AHSG < 365 μg/ml**	**AHSG ≥ 365 μg/ml**
1 month	0.500 ± 0.129	0.000 ± 0.000
3 months	0.897 ± 0.222	0.077 ± 0.384
6 months	0.909 ± 0.362	0.020 ± 0.000
12 months and after*	0.943 ± 0.420	0.000 ± 0.000

* Since the observation period was limited to 12 months hazard rate at this time interval should be considered as an interpolation. Hazard rate: mean ± SE

**Table 5:** Association of low fetuin A/α 2HS-glycoprotein levels with 1-year mortality of patients with alcoholic liver cirrhosis calculated by adjusted multiple logistic regression*

	**(A) 1-year follow-up period**		**(B) Follow-up between months 1–12**	
**Variable**	p*	p*	p*	p*

Hematocrit	0.482	0.597	0.463	0.453
Serum albumin	0.271	0.832	0.249	0.994
Transferrin	0.706	0.838	0.608	0.843
Serum creatinine	-	0.633	-	0.806
INR	-	0.290	-	0.262
MELD < 20, ≥ 20	0.172	-	0.165	-
AHSG < 365 μg/ml, ≥ 365 μg/ml	0.004	0.010	0.021	0.012
Odds Ratio of AHSG < 365 μg/ml, ≥ 365 μg/ml	58.82 95% C.I.: 3.703 – 950.8	49.35 95% C.I.: 2.497 – 975.6	28.91 95% C.I: 1.661 – 503.4	32.59 95% C.I.: 1.502 – 707.0
Model fit	p = 0.015	p = 0.068	p = 0.002	p = 0.012

* P value of regression coefficient, adjusted for variables significantly associated with mortality at univariate analysis (Table 1)

**Table 6:** Association of serum AHSG concentration, the CTP and MELD scores with mortality of patients with alcoholic liver cirrhosis calculated by adjusted multiple logistic regression

	**1-year follow-up**		**Months 1–12**	
**Variable**	p*	**OR 95% C.I.**	p*	**OR 95% C.I.**

CTP score < 10, ≥ 10	0.004	17.54	0.005	15.58
		2.556 – 119.9		2.333 – 104.1
MELD score < 20, ≥ 20	0.110	-	0.120	-
AHSG < 365 μg/ml, ≥ 365	< 0.001	170.6	< 0.001	89.43
μg/ml		22.73 – 1280		11.89 – 672.6
Model fit	p = 0.114		p = 0.001	

*: p value of regression coefficient
